# High Bubble Grade After Diving: The Role of the Blood Pressure Regimen

**DOI:** 10.3389/fphys.2019.00749

**Published:** 2019-06-20

**Authors:** Alain Boussuges, Guillaume Chaumet, Nicolas Vallée, Jean Jacques Risso, Jean Michel Pontier

**Affiliations:** ^1^ ERRSO, Institut de Recherche Biomédicale des Armées (IRBA), Toulon, France; ^2^ Center for Cardiovascular and Nutrition Research (C2VN), INSERM, INRA, Aix Marseille Université, Marseille, France; ^3^ Altra Bio SA, Lyon, France; ^4^ Cephismer, Centre d’expertise plongée pour la Marine Nationale, Toulon, France

**Keywords:** circulatory system, decompression, endothelial function, SCUBA diving, venous gas emboli

## Abstract

**Introduction:** Previous studies have suggested that the circulatory system was involved in the production of circulatory bubbles after diving. This study was designed to research the cardio-vascular function characteristics related to the production of high bubble grades after diving.

**Methods:** Thirty trained divers were investigated both at baseline and after a 30-msw SCUBA dive. At baseline, the investigations included blood pressure measurement, echocardiography, and assessment of aerobic fitness using VO_2_ peak measurement. Blood samples were taken at rest, to measure the plasma concentration of NOx and endothelin-1. After diving, circulating bubbles were detected in the pulmonary artery by pulsed Doppler at 20-min intervals during the 90 min after surfacing. The global bubble quantity production was estimated by the KISS index.

**Results:** Divers with a high bubble grade (KISS > 7.5) had systolic blood pressure, pulse pressure, weight, and height significantly higher than divers with a low bubble grade. By contrast, total arterial compliance, plasma NOx level, and percentage of predicted value of peak oxygen uptake were significantly lower in divers with a high bubble grade. Cardiac dimensions, left ventricular function, and plasma endothelin-1 concentration were not significantly different between groups. The multivariate analysis identified blood pressure as the main contributor of the quantity of bubble production. The model including pulse pressure, plasma NOx level, and percentage of predicted value of peak oxygen uptake has an explanatory power of 49.22%.

**Conclusion:** The viscoelastic properties of the arterial tree appeared to be an important contributor to the circulating bubble production after a dive.

During the decompression stage after a dive, circulating bubbles are commonly observed in the venous system. Since the 1970s, venous gas emboli (VGE) can be screened by ultrasonography and Doppler. To quantify the number of bubbles, it is usual to rate the number and frequency of bubbles compared with the heartbeat. Using this method, bubble grades have been proposed (Spencer, 1976; Kisman et al., 1978; Boussuges et al., 1998). Previous studies have researched the correlation between the venous bubble quantity and the probability of decompression sickness (Nashimoto and Gotoh, 1978; Nishi et al., 1981; Eatock, 1984; Bayne et al., 1985). The correlation is debatable, but all authors have observed that, when the quantity of circulating bubbles is low, the risk is also low. Consequently, bubble screening has been used as a safety indicator for diving profiles (Eatock and Nishi, 1987). A decompression profile producing a small quantity of circulating bubbles in a large population of divers is recognized as a low risk dive. A conservative decompression profile is particularly important in divers with patent foramen ovale (Boussuges et al., 2014; Honek et al., 2014). Furthermore, a wide inter-individual susceptibility to bubble formation has been reported (Papadopoulou et al., 2018). Indeed, for the same given dive profile, no circulating bubbles were detected in some divers whereas other individuals presented a large quantity of circulating bubbles. The mechanism explaining this variability remains largely unknown. VGE are made up from pre-existing gas nuclei and enriched by the neutral gas in the breathing mixture, which is in a supersaturated state in blood and tissues, when the ambient pressure drops. The sites and mechanisms for the formation of gas nuclei remain uncertain (Blatteau et al., 2006). Some authors have suggested that gas nuclei might be trapped in hydrophobic crevices. These hydrophobic sites might be present on the surface of the endothelium in the form of caveolae (Brubakk, 2004). The impact of vasomotor tone and endothelial function on bubble formation has also been supported by previous studies (Dujic et al., 2006). An increase in nitric oxide (NO)-dependent relaxation leads to a decrease in bubble grade (Wisløff et al., 2004). In contrast to this finding, in animals subjected to a hyperbaric exposure, an inhibition of NO induced both an increase in bubble grade and mortality (Wisløff et al., 2003). Lastly, in volunteers, Cialoni et al. reported an increase in nitric oxide levels during a SCUBA dive (Cialoni et al., 2019), suggesting an endothelial stimulation at the bottom.

According to the previous studies, we hypothesized that the viscoelastic properties of the circulatory system of healthy volunteers studied at baseline before a dive, differed between bubble-prone divers and bubble-resistant divers.

## Materials and Methods

All the procedures were conducted in accordance with the Declaration of Helsinki and were approved by the local Ethics Committee (CCPPRB 1 Aix Marseille No. 20062103). Each method and the potential risks were explained to the participants in detail, and they gave written informed consent before the experiment.

### Subjects

Thirty trained male divers, aged 37 ± 7 years, weight 77 ± 8 kg, height 175 ± 8 cm, BMI 25 ± 2 kg m^–2^, and body surface area 1.93 ± 0.14 m^2^, volunteered to participate in this experiment. All of them were regular recreational or professional SCUBA divers with 100–4,000 dives and without a past history of diving injury. Each participant underwent a physical examination and a full medical history. The subjects were non-smokers and were included if they had no hypertension, cardiovascular or kidney disease, and no medication during the study.

### Baseline Investigations

During the inclusion visit, it was verified that volunteers had a similar arterial pressure on both upper limbs.

#### Assessment of Aerobic Fitness

To assess individual aerobic fitness, each volunteer performed an incremental fatigue treadmill test. Ventilatory and gas-exchange parameters were measured using a breath-by-breath system (Cosmed Quark PFT ergo, Rome, Italy) which was calibrated before each test. The subjects spent 3 min at rest to reach a steady-state gas exchange condition. Thereafter, all participants carried out a 4-min warm-up running session at 8 km h^−1^ with an elevation of 2%, after which the treadmill speed was increased by 1 km h^−1^ every 1 min until volitional fatigue was reached. The data were averaged for 20 s and the VO_2_ peak was defined as the highest value of oxygen uptake despite increased workload. The criteria indicating maximal exercise were as follows: plateauing of oxygen consumption, respiratory gas exchange ratio ≥1.1, and heart rate (HR) ≥95% age-predicted maximal HR. The results were reported in VO_2_ peak (ml kg^−1^ min^−1^) and in percent-predicted peak VO_2_ calculated according to the equation [weight (kg) × 56.36 − (0.413 × age)] proposed by Wasserman et al. (1987). No diver was considered to be overweight, according to the formula: weight (kg) > 0.79 × height (cm) − 60.7.

#### Echocardiographic Study

Divers underwent the echocardiographic examinations in basal conditions 1 h before the dives. The subjects were placed in left lateral decubitus. HR was recorded by echocardiogram and the rate was averaged over 60 s. The cardiac ultrasound examinations were carried out by an experienced investigator (AB) using a commercially available echocardiograph (Mylab 25, Genoa, Italy) connected to a transducer array of 2.5–3.5 MHz. At this time, any cardiac abnormalities resulted in the exclusion of the subject from the study. Doppler recordings were performed at the end of a normal expiration in order to eliminate the effects of respiration on the parameters studied. Measurements were averaged from at least three different beats.

##### Left Heart Study

Left atrial (LA) diameter, left ventricle (LV) end systolic and end diastolic diameters (ESD, EDD), left ventricle end systolic and end diastolic interventricular septal thickness, and left ventricle end systolic and end diastolic posterior wall thickness were measured by M-mode echocardiography from the left short and long axis views. Left ventricular mass (LVM) was assessed by M mode echocardiography and the application of Devereux’s formula (Devereux and Reichek, 1977). The standard index of global LV systolic performance was LV percent fractional shortening (%FS) as the ratio (LV EDD − LV ESD)/LV EDD.

Left ventricular filling was studied using transmitral blood flow velocities recorded by pulsed Doppler. Transmitral blood flow velocities were obtained from the apical four-chamber view, positioning the sample volume at the mitral valve leaflet tips. Doppler velocity curves were recorded at 100 mm s^−1^. Peak velocity and velocity-time integral (VTI) of the initial flow (E wave), representing the early filling phase, and of the late flow (A wave), representing the atrial contraction, were measured. The peak velocities ratio (*E*/*A*) and the ratio of the A wave VTI to the total VTI (relative contribution of atrial contraction to the total LV filling) were calculated. The interval from the aortic valve closure signal to the mitral valve opening signal (IVRT) was also measured.

Tissue Doppler imaging (TDI) of the mitral annulus during diastole was recorded. The ratio of transmitral early diastolic velocity (*E*) to TDI early diastolic velocity of the mitral annulus (*E*′) was calculated as an index of LV filling pressures (Nagueh et al., 1997).

##### Right Heart Study

Right ventricle end-diastolic diameter (RVEDD) was measured by M-mode echocardiography from the left parasternal long axis views. The measurement of the peak of the tricuspid regurgitation velocity (TRV) was performed using continuous wave Doppler.

The RV outflow tract time-velocity integral (TVI_RVOT_) was recorded from the parasternal short axis view. Pulmonary vascular resistance (PVR) was estimated by the formula [(TRV/ TVI_RVOT_ × 10) + 0.16] in Wood units (WU) according to the method proposed by Abbas et al. (2003). Furthermore, the acceleration time/RV ejection time ratio of the pulmonary artery blood flow (AcT/RVET) was calculated to assess the pulmonary artery pressure (PAP) regimen owing to the negative correlation between the AcT/RVET and the mean PAP (Kitabatake et al., 1983). The inferior vena cava diameter was measured at the end of the expiration from a subcostal view.

##### Hemodynamic Data

Cardiac output (CO) was derived from the aortic blood flow. The aortic cross-sectional diameter was measured by 2D echocardiography from the left parasternal short axis view at the level of the aortic root. Aortic cross-sectional area (ACSA) was calculated as: ACSA = 3.14 × *d*^2^/4.

The aortic systolic flow velocity -time integral (VTI Ao) was measured using the pulsed wave Doppler profile of aortic blood flow from the apical four chamber view making it possible to calculate LV stroke volume (LV SV = VTI Ao × ACSA) and cardiac output (LV CO = LV SV × HR).

Systemic vascular resistance and total arterial compliance were calculated as mean arterial pressure/CO and LV SV/pulse pressure (PP), respectively (Chemla et al., 1998).

#### Blood Pressure Measurement

Sphygmomanometric blood pressure measurements on the right arm were obtained using an automatic device (Omron HEM-705CP, Bannockburn, IL, USA) at the end of each echocardiographic examination. This automated device was validated by the British Hypertension Society and the Association for the Advancement of Medical Instrumentation (Asmar and Zanchetti, 2000).

#### Biological Study

Blood samples were taken before the dives. The tubes were immediately placed in iced water and centrifuged for 15 min at 4,000 rmin^−1^ at 4°C. Plasma was then stored at −70°C until analysis. Nitric oxide (NO) is rapidly converted in plasma; consequently to estimate NO bioavailability, the sum of stable metabolites of NO, i.e., nitrate and nitrite (NOx) was used. Plasma NOx was determined using a spectrophotometric kit (Cayman Chemical, Ann Arbor, MI, USA). Plasma concentration of endothelin-1 was measured using a commercially available ELISA kit (R&D Systems, Minneapolis, MN, USA).

### Dives

The day before the dives it was recommended that no intense physical activity was performed and a low-nitrate diet was followed. All the dives were performed 3–4 h after a light meal between 10 and 11 a.m. The volunteers were weighed both before and after the dives. The divers breathed air during the dives. They were equipped with neoprene diving wet suits, the thickness of which was in accordance with the temperature of the sea (from 15 to 20°C at the surface). The dive profile was the same for all divers. They performed the SCUBA dives in open seawater at 30 m of seawater (msw) on a regular flat bottom. The bottom time including the descent time (velocity of the descent time from 10 to 15 msw min^−1^) was 30 min. At the bottom, the divers performed a regular finning action for a distance to 400 m. After 30 min, the ascent rate up to the decompression stop was 9–10 msw min^−1^. Decompression stop (9 min at 3 msw) was in accordance with the French Navy procedure (Marine Nationale 90). After the dives, they were instructed to reduce activity as much as possible.

### Examinations After the Dives

The duration of the transfer to the laboratory was around 25 min (the return of the boat to port and the route from the port to the laboratory); consequently, the investigations began 30 min after the end of the dive. Examiners were blinded to the results of the other investigations (previous examinations or investigations performed by the other examiners). Investigations were undertaken in a quiet room with a controlled environmental temperature (28°C).

#### Circulating Bubble Detection

Circulating vascular bubble detections were performed by an experienced investigator (JMP) using a pulsed Doppler equipped with a 2-MHz probe (Pioneer-Siemens, Malvern, USA). The screening tests were performed in the laboratory at 20-min intervals during the 90 min after surfacing (30, 50, 70, and 90 min). The subjects were in the left lateral position and rested for 1 min before the test. The Doppler probe was placed along the left edge of the sternum to record the pulmonary artery blood flow. VGE were monitored in the pericardial area with the divers at rest, during muscle contraction of the quadriceps (the patients were told to contract the quadriceps to pull the patella superiorly tightly during 10 s) and during flexion of the lower limb (hip and knee flexion to 90°). Each maneuver was repeated twice. The Spencer scale was used to quantify the bubble amount (Spencer, 1976). The quality of the screening was assessed for each recording and rated as satisfactory, average, or poor. The screening sessions were recorded and then analyzed by two independent investigators. If any discrepancy in the interpretation of the signals occurred, the recording was studied again in order to reach a consensus. When a consensus was lacking the diver was excluded from the study.

Furthermore, after diving, an echocardiographic examination has been repeated 1 h after emersion. During this examination, it was performed both the study of the cardiac function (results not reported in the present work) and the assessment of the circulating bubbles quantity.

The Kisman-Masurel integrated severity score (KISS) was calculated from bubble detections performed at rest, to estimate the magnitude of the evolved gas phase induced by the decompression stress (Nishi et al., 1981).

Thereafter, the population was divided in two groups according to their bubble production:

- Divers with a low bubble grade (LBG) producing few bubbles, i.e., with a maximal bubble grade ≤2 and a KISS index <7.5.
- Divers with a high bubble grade (HBG) producing an important circulating bubble quantity, i.e., with a maximal bubble grade >2 and a KISS index >7.5.

### Statistical Analysis

Data are expressed as mean ± standard deviation. All the statistical analyses were performed with R statistical software (Lagani et al., 2016). The distribution of the variables was studied by a Kolmogorov-Smirnov test. To compare the characteristics of the cardio-vascular function in divers with HBG and LBG, a t-test was used. When the variables were not normally distributed a Mann-Whitney test was performed. Significance was *p* < 0.05.

We then searched for the factors associated with the bubble production assessed by the KISS index. First, the relationship between the KISS index and variables was described by using Spearman’s correlation. Second, we selected the set of variables that could explain the high bubble grade, using a logistic regression model. Because we suspected multicollinearity between our explanatory variables, we excluded variables with the highest variance inflation factor (VIF) *via* a stepwise method: the algorithm calculates the VIF score for all explanatory variables and removes the variable with the highest value, then recalculates the VIF scores on the remaining values, until there are no variables with a VIF greater than the threshold (10 here) (Naimi et al., 2014). On these selected variables, we then used the variable selection method. Among the various methods of variable selection, we chose the adaptation for logistic regression (Lozano et al., 2011) of the generalized orthogonal matching pursuit algorithm (Pati et al., 1993). This algorithm was implemented in R (Lagani et al., 2016).

## Results

### Baseline Investigations

All divers had a normal left ventricular systolic and diastolic function. Left ventricular mass was measured as a mean of 198 ± 52 g (104 ± 27 g m^−2^). The quality of the echocardiographic examinations was considered to be poor in two divers. These volunteers were excluded from the subsequent analysis. VO_2_ peak was measured as a mean of 49 ± 6 ml kg^−1^ min^−1^ (120 ± 14% of the predicted value).

Plasma NOx level was 30 ± 8 μmol L^−1^. Plasma endothelin-1 concentration was 1.4 ± 0.7 pg ml^−1^.

### Examinations After the Dives

None of the divers presented any disorders suggesting a decompression accident.

#### Study of the Whole Population

Significant weight loss was found after the dives (as a mean 590 ± 390 g – 0.8% of body mass).

One hour after the dive, the quantity of circulating bubbles assessed by pulsed Doppler and echocardiography were closely related.

**Figure 1:**
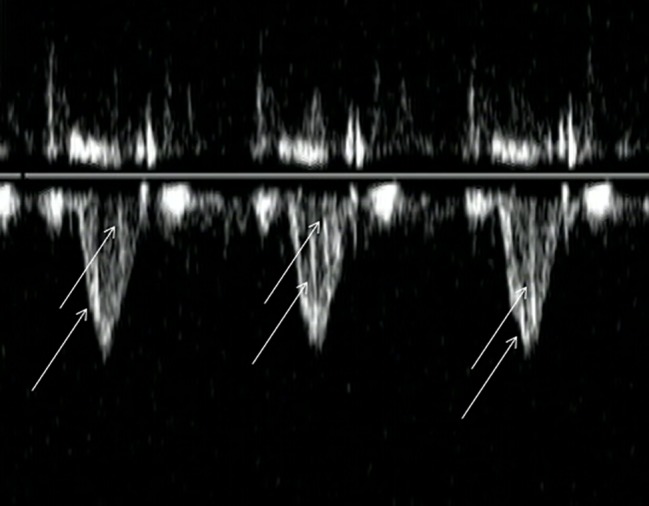
Circulating bubbles detected in the pulmonary artery blood flow: the arrows indicate bubble signals in each cycle (Grade 3: the majority of the cardiac periods contain bubble signals singularly or in group).

**Figure 2:**
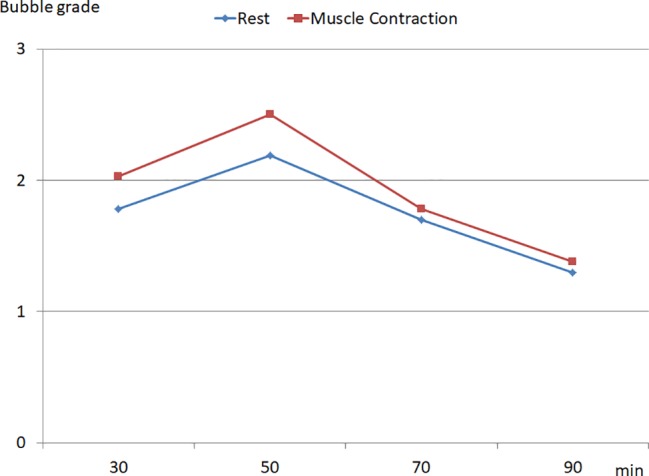
Time course of the bubble grade recorded after the dive, at rest and during muscle contraction of the lower limbs (data are expressed as median).

A significant inter-individual variability in the production of VGE was observed in the population. In some divers (*n* = 3), no bubbles were detected whereas in other individuals Grade 3 (Figure 1) was recorded over the whole detection period. Mean KISS index was calculated as 17.4 ± 16 (ranging from 0 to 42). The muscle contractions of the lower limbs led to an increase in circulating bubbles (Figure 2).

According to the criteria previously mentioned in section “Methods,” the population was divided into divers with a high bubble grade (13 divers) and divers with a low bubble grade (15 divers).

#### Comparison Between Divers With an HBG and Divers With an LBG

The mean age of the two groups of divers with an LBG and an HBG was not significantly different (38 ± 6 vs. 36 ± 7 years, respectively).

Divers with an HBG had a weight (80 ± 7 vs. 73 ± 9 kg – *p* < 0.05) and a height (179 ± 6 vs. 172 ± 7 cm – p < 0.05) significantly higher than divers with an LBG. In total, the body surface area of the divers with an HBG was larger than divers with an LBG (1.99 ± 0.1 vs. 1.86 ± 0.14 m^2^). BMI was not significantly different between the two groups (25 ± 1.9 vs. 24.9 ± 1.8 kg m^−2^).

The VO_2_ peak was not significantly different between groups (LBG divers 50 ± 6 vs. HBG divers 48 ± 7 ml kg^−1^ min^−1^). Nevertheless, when the result was expressed as the percentage of the predicted value [according to the equation proposed by Wasserman et al. (1987)], the difference reached significance (LBG divers: 126 ± 13% vs. HBG divers: 115 ± 14% of the predicted value – *p* < 0.05).

Echocardiographic parameters including left ventricular systolic and diastolic function and right heart study were not significantly different between the two groups (Tables 1 and 2).

**Table 1:** Left ventricular systolic and diastolic function.

	Divers with an LBG	Divers with an HBG	
LA (mm)	35 ± 4	34 ± 3	NS
LV EDD (mm)	53 ± 3	54 ± 2	NS
LV ESD (mm)	33 ± 3	34 ± 3	NS
LV mass/BSA (g m^−2^)	104 ± 31	103 ± 21	NS
LV FS (%)	38 ± 5	36 ± 4	NS
E (cm s^−1^)	70 ± 17	71 ± 11	NS
A (cm s^−1^)	46 ± 9	49 ± 16	NS
E/A ratio	1.6 ± 0.4	1.6 ± 0.6	NS
IVRT (ms)	74 ± 8	76 ± 8	NS
LA contribution (%)	25 ± 6	26 ± 8	NS
*E*′ (cm s^−1^)	16 ± 3	16 ± 3	NS
*E*/*E*′ ratio	5 ± 2	5 ± 1	NS

*LBG, low bubble grade; HBG, high bubble grade; LA, left atrium; LV, left ventricle; EDD, end diastolic diameter; ESD, end systolic diameter; BSA, body surface area; LV FS, left ventricle fraction shortening; E, peak velocity of the initial trans mitral flow; A, peak velocity of the late flow; IVRT, isovolumetric relaxation time; LA contribution, contribution of atrial contraction to the LV filling; E΄, early diastolic velocity of the mitral annulus*.

**Table 2:** Right heart study.

	Divers with an LBG	Divers with an HBG	
IVC (mm)	19 ± 3	21 ± 5	NS
RVEDD (mm)	22 ± 1	21 ± 3	NS
TRV (cm s^−1^)	2 ± 0.2	2 ± 0.1	NS
PVR (WU)	0.1 ± 0.02	0.1 ± 0.02	NS
AcT/RVET (%)	47 ± 4	47 ± 6	NS

*LBG, low bubble grade; HBG, high bubble grade; IVC, diameter of the inferior vena cava at expiration; RVEDD, right ventricle end-diastolic diameter; TRV, peak of the tricuspid regurgitation velocity; PVR, pulmonary vascular resistance; WU, Woods unit; AcT/RVET, acceleration time/RV ejection time ratio of the pulmonary artery blood flow*.

Hemodynamic data did not report differences in HR and cardiac output between groups (Table 3). However, brachial blood pressure was different between the two groups. Systolic arterial pressure and pulse pressure were significantly higher in divers with an HBG when compared with LBG divers. Total arterial compliance estimated by the ratio SV/PP was lower in divers with an HBG when compared with divers with an LBG.

**Table 3:** Hemodynamic data.

	Divers with an LBG	Divers with an HBG	
SBP (mmHg)	118 ± 7	126 ± 7	*p* < 0.01
DBP (mmHg)	70 ± 7	71 ± 6	NS
MBP (mmHg)	86 ± 6	90 ± 6	NS
PP (mmHg)	48 ± 6	54 ± 6	P < 0.01
Heart rate (beat min^−1^)	65 ± 7	65 ± 8	NS
Ao VTI (cm)	21 ± 3	20 ± 2	NS
SV (ml)	90 ± 16	89 ± 10	NS
Cardiac output (L min^−1^)	5.8 ± 1.1	5.8 ± 1	NS
SVR (dyne s^−1^ cm^−1^)	1,236 ± 250	1,260 ± 156	NS
SV/PP (ml mmHg^−1^)	1.9 ± 0.3	1.6 ± 0.3	<0.05

*LBG, low bubble grade; HBG, high bubble grade; SBP, systolic blood pressure; DBP, diastolic blood pressure; MBP, mean blood pressure; PP, pulse pressure; Ao VTI, velocity-time integral of the aortic blood flow; SV, stroke volume; SVR, systemic vascular resistance; SV/PP, total arterial compliance*.

#### Biological Study

The plasma endothelin-1 concentration was not significantly different between the two groups (divers with an LBG 1.2 ± 0.1 pg ml^−1^ vs. divers with an HBG 1.7 ± 1 pg ml^−1^).

Plasma NOx level was higher in divers with an LBG (34.5 ± 6 μmol L^−1^) when compared with divers with an HBG (23.3 ± 4 μmol L^−1^ – *p* < 0.05).

#### Results of the Multivariate Analysis

**Figure 3:**
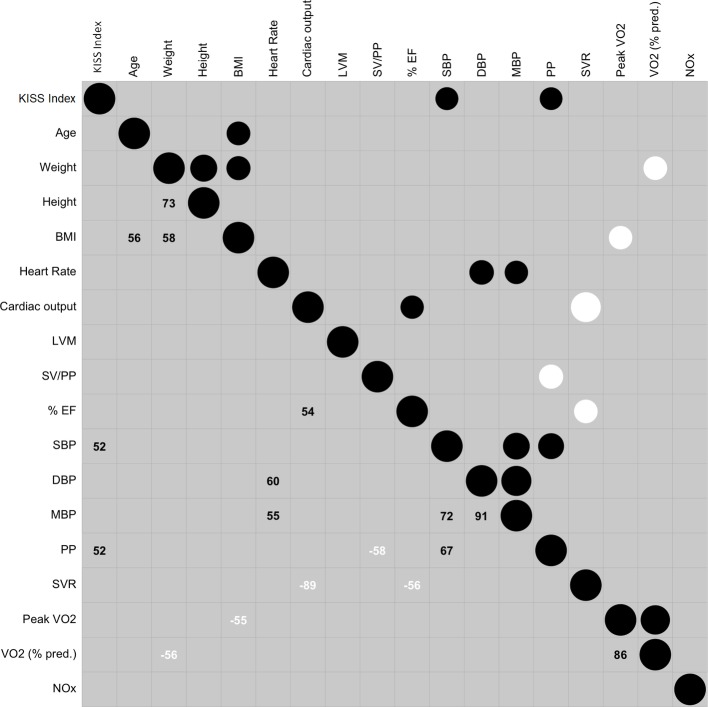
Correlogram reporting the relationship between the variables. The circles in the upper part contain two information: the size reports the absolute value of the relation: the bigger the size is, the higher the strength is. Positive relationship is in black and negative relationship is in white. The larger number in the lower part is r in percentage (*r* × 100). Abbreviations: KISS, Kisman-Masurel integrated severity score; BMI, body mass index; LVM, left ventricular mass; SV, stroke volume; SV/PP, total arterial compliance; LV FS, left ventricle fraction shortening (%EF); SBP, systolic blood pressure; DBP, diastolic blood pressure; MBP, mean blood pressure; PP, pulse pressure; SVR, systemic vascular resistance; peak VO_2_, highest value of oxygen uptake; VO_2_ (% pred.), percentage of predicted peak VO_2_; NOx, metabolites of nitric oxide.

Correlation result was represented in the correlogram (Figure 3).

The variable inflation factor step procedure kept the following variables: age, height, BMI, HR, left ventricular mass, % EF, PP, SVR, VO_2_ (% predicted), and plasma NOx level.

The generalized orthogonal pursuit algorithm selected the following model: PP, VO_2_ (% predicted, and plasma NOx level with the following results: this model had an explanatory power of 49.22%. The model’s intercept was at 3.26 (SE = 6.83, 95% CI [−9.63, 18.75]). Within this model:

- The effect of PP was significant (beta = 0.23, SE = 0.095, 95% CI [0.067, 0.45], *z* = 2.38, *p* = 0.0171) and can be considered as large (std. beta = 1.56, std. SE = 0.65).
- The effect of VO_2_ (% predicted) tended to be significant (beta = −0.093, SE = 0.052, 95% CI [−0.22, −0.0094], *z* = −1.79, *p* = 0.07) and could be considered as medium (std. beta = −1.31, std. SE = 0.73).
- The effect of the plasma NOx level was not significant (beta = −0.12, SE = 0.088, 95% CI [−0.36, −0.0053], *z* = −1.34, *p* = 0.1815) and could be considered as medium (std. beta = −1.32, std. SE = 0.99).

The Likelihood ratio test result of PP + VO_2_ (% predicted) + plasma NOx level model was the following log Lik = −19.337 (Df = −3), Chisq = 17.071, *p* = 0.0006833.

As expected, both correlogram and VIF algorithm results showed that most of the hemodynamic and blood pressure variables were correlated and collinear. In the final model, we observed that plasma NOx was not significantly related to the high bubble grade state but when plasma NOx variable was dropped, the new model had a decrease of the explanatory power (from 49.2 to 38.52%).

## Discussion

The main result of this study was that volunteers with an HBG after the dive presented both higher SBP and PP at baseline when compared with divers with an LBG. The multivariate analysis supported this finding. Indeed, the main contributor to the bubble quantity assessed by the KISS index was the blood pressure regimen.

It is recognized that the determinants of systolic blood pressure are stroke volume, HR, and the distensibility of the arterial tree (Smulyan and Safar, 1997). In our study, since there was no difference in cardiac output, stroke volume, and HR between groups, the higher SBP in divers with an HBG could be attributed to a reduced arterial distensibility in this group. This interpretation was supported by the wider pulse pressure recorded in divers with an HBG. Indeed, it has been demonstrated that an increased stiffness of the central arteries led to an increase in PP through an impairment in arterial compliance and an increase of the impact of the wave reflection on the blood pressure (Dart and Kingwell, 2001). Some anthropometric characteristics are recognized as risk factors for decompression sickness and HBG after diving (Cialoni et al., 2017). In our population, anthropometric data reported differences between groups. Divers with an HBG had a larger body surface area than divers with an LBG. It is recognized that the intensity of arterial wave reflections are positively correlated with height (Asmar et al., 1997). Furthermore, it has been reported a significant positive correlation between systolic blood pressure and body size (weight, height, or BSA) in healthy young adults (Evans et al., 2017). Consequently, the difference in body size could have contributed to the higher pulse pressure in divers with an HBG.

Estimating the total arterial compliance by the ratio stroke volume divided by pulse pressure (Chemla et al., 1998) has been proposed. When compared with divers with an LBG, SV/PP was significantly lower in divers with an HBG supporting an increased stiffness of the central arteries in this group. Arterial compliance varies according to several factors such as age, physical fitness, and pathophysiological states such as hypercholesterolemia, diabetes, and hypertension. In this study, the divers were healthy non-smoker volunteers, and divers with an HBG had a similar age and VO_2_ peak to divers with an LBG. Nevertheless, the percentage of predicted VO_2_ peak was significantly higher in divers with an LBG, suggesting that aerobic fitness was higher in this group when compared with divers with an HBG. This result was supported by the multivariate analysis. Indeed, the percentage of predicted VO_2_ peak was retained in the model. The impact of aerobic fitness on the production of VGE after a dive has been previously reported by some studies (Carturan et al., 2000; Schellart et al., 2012). Furthermore, it has been demonstrated that aerobic exercise training, was able to improve central arterial compliance in middle-aged men (Hayashi et al., 2005). Consequently, the physiological mechanism explaining the impact of aerobic fitness on VGE production after a dive might be the viscoelastic properties of the vascular tree. Endothelial function is implicated in the improvement in arterial compliance induced by aerobic exercise training. A single bout of physical exercise leads to an elevation in cardiac output, pulse pressure, and arterial blood flow and subsequently to an increase in laminar shear stress on vessel walls. Repetitive endothelial stressors induced by endurance physical training are able to increase plasma nitric oxide (NO) concentration and NO bioavailability (Green et al., 2004; Higashi and Yoshizumi, 2004; Rush et al., 2005; Goto et al., 2007). In our work, the results for the plasma NOx level agreed with these previous studies. Indeed, the plasma NOx level was higher in divers with an LBG, suggesting a higher NO bioavailability in this group. In the multivariate analysis, the effect of plasma NOx level on the bubble production was considered as medium: the integration of plasma NOx level in the model improved the explanatory power from 38.5 to 49.2%. The main contributor of high bubble grade was the blood pressure regimen.

A number of factors are known to influence arterial wall behavior and, therefore, blood pressure. Arterial compliance varies with the physical properties of the arterial media which contains smooth muscle cells, elastin, and collagen. Smooth muscle tone is affected by nervous activity, by hormones, and by locally produced vasoactive substances. Endothelial cells produce several important vasoactive substances including nitric oxide, and other factors such as prostacyclin, endothelium-derived hyperpolarizing factor, carbon monoxide, endothelin, and vasoactive prostanoids. The number of factors affecting the mechanical properties of the arterial wall explains that arterial compliance cannot be assessed by the sole measurement of NO production.

A negative relationship has been reported between endothelin-1 and aerobic physical fitness (Otsuki et al., 2006). Nevertheless, in our population, a significant difference in plasma endothelin-1 concentration in divers with an LBG and an HBG was not found.

In total, the viscoelastic properties of the arterial tree appeared to be an important contributor to circulating bubble production after a dive. This finding makes it possible to explain the results of some previously published studies.

It has been observed that older divers produced more bubbles than younger divers (Carturan et al., 2002; Schellart et al., 2012). This result could be attributed to the decrease in arterial compliance commonly observed with increasing age.

Higher NO biosynthesis has been reported in pre-menopausal women when compared with men (Forte et al., 1998). This difference is probably related to sex hormones (Cicinelli et al., 1996) because NO production decreases after menopause. After diving, less bubble production in premenopausal women has been observed in comparison with postmenopausal women and male divers (Boussuges et al., 2009).

Lastly, it has been demonstrated that a single bout of aerobic exercise reduced central and peripheral blood pressure and increased arterial compliance (Kingwell et al., 1997). Post-exercise hypotension can persist for several hours. The impact of exercise on the arterial tree might explain why an endurance exercise performed many hours before a dive could decrease the circulating bubble quantity after the dive (Dujic et al., 2004; Blatteau et al., 2007).

### Study Limits and Hypotheses

The interpretation of the present study should be cautious: indeed some study limits can be stressed.

The interest of plasma NOx concentration in assessing NO bioavailability has been questioned (Kim-Shapiro and Gladwin, 2015). Nevertheless, in our work, the contribution of the arterial function in venous gas bubble production after the dive was supported by several independent or combined data such as blood pressure measurements, ultrasonography study (assessment of total arterial compliance) and biological parameters (plasma NOx concentration).

The method of bubble detection followed the guidelines (Mollerlokken et al., 2016). To improve the assessment of the circulating bubbles quantity, we have used repeated detections. Screening Doppler tests began 30 min after the dive and were conducted every 20 min for 90 min. The KISS index was used as a semi quantitative method to estimate the whole bubble production. After a similar dive profile, the maximal bubble grade has been assessed to be around 40–50 min (Boussuges et al., 2009). On the other hand, VGE can be detected for several hours after diving (Masurel et al., 1976). To assess the total bubble activity prolonged monitoring would be better. Furthermore, to distinguish bubble-prone divers and bubble-resistant divers, we have chosen to separate the population in two groups; divers with LBG, i.e., with bubble grades 0, 1, or 2 leading to a KISS index <7.5 and divers with HBG, i.e., with bubble grades 3 or 4 with a KISS index >7.5. In further studies, for a better assessment of the whole bubble production for each diver, the use of two dimensional echocardiography combined with a computerized automatic counting of the circulating bubbles would be advantageous (Germonpré et al., 2014).

Lastly, in this study some factors known to impact bubble production such as age and BMI were not observed (Carturan et al., 2002). Our study included professional or recreational divers with good physical fitness (VO_2_ peak from 36 to 62 ml kg^−1^ min^−1^). None of the divers were overweight. Furthermore, most divers were middle-aged (24 out of 30 were from 30 and 45 years). A larger range for age and BMI could be more appropriate to study these factors.

The mechanism explaining the impact of arterial compliance on the circulating bubbles quantity remains hypothetical. The visco-elastic properties of the arterial wall might affect the diffusion of inert gas. Indeed, it has been reported that the systemic infusion of an endogenous nitric oxide synthase inhibitor lead to an increase in vascular stiffness and a decrease in cerebral blood flow in healthy subjects (Kielstein et al., 2006). As suggested by Arieli and Marmur (2017), the reduction of peripheral blood flow, in divers with low arterial compliance, might enhance the diffusion of inert gas into the artery and therefore the expansion of microbubbles.

## Conclusion

In the present study, arterial compliance appeared to be a major factor in the circulating bubble quantity after a dive. The measurement of blood pressure including pulse pressure can provide the dive medical doctors with a simple means of estimating the risk of a high quantity of circulating bubbles after diving. It is therefore important to check blood pressure regularly in professional divers. When an increase in systolic blood pressure and pulse pressure is recorded, it should be recommended that dietary changes are made and the duration of endurance exercise training increased. These recommendations can be beneficial for reducing both the cardio-vascular risk factors and the quantity of bubble production after diving.

## Data Availability

The raw data supporting the conclusions of this manuscript will be made available by the authors, without undue reservation, to any qualified researcher.

## Ethics Statement

All the procedures were conducted in accordance with the Declaration of Helsinki and were approved by the local Ethics Committee (CCPPRB 1 Aix Marseille No. 20062103). Each method and the potential risks were explained to the participants in detail and they gave written informed consent before the experiment.

## Author Contributions

AB and JP conceived and designed the study. NV and JR assisted with the technical aspects of the protocol, recruited all the participants, and were involved in the acquisition of the data. AB and GC analyzed the data and performed the statistical analysis. AB, GC, and JP have drafted the article, while NV and JR revised it critically for important intellectual content. All the authors have given final approval of the version to be published.

### Conflict of Interest Statement

The authors declare that the research was conducted in the absence of any commercial or financial relationships that could be construed as a potential conflict of interest.
